# A practical guide for characterization of novel CRISPR-Cas systems with Pro-CRISPR factors

**DOI:** 10.52601/bpr.2025.240066

**Published:** 2026-04-30

**Authors:** Ao Sun, Shu-Lin Jin, Jun-Jie Gogo Liu

**Affiliations:** 1 Beijing Frontier Research Center for Biological Structure, Beijing Advanced Innovation Center for Structural Biology, State Key Laboratory of Membrane Biology, Tsinghua-Peking Center for Life Sciences, School of Life Sciences, Tsinghua University, Beijing 100084, China

**Keywords:** CRISPR-Cas system, Genome editing, Cas nuclease, Pro-CRISPR, Protein purification, Biochemical properties

## Abstract

The emergence of advanced genome editing technologies has revolutionized research in life sciences, offering an unprecedented way to uncover unknown biological functions and innovative therapeutic strategies. Among all genome editing tools, CRISPR-Cas-based technologies play a pivotal role in this revolution, particularly Class 2 effectors such as Cas9 and Cas12, owing to their high efficacy and ease of programmability. With the advancements in genome sequencing and metagenomics, an increasing number of novel CRISPR-Cas systems have been discovered, including those found in extreme environments and viruses. Furthermore, recent studies have revealed an unexpected role of non-Cas accessory genes, such as the Tn7-like transposon and Pro-CRISPR factors (Pcr), in conferring additional functionalities to the CRISPR system, providing new insights into the understanding of CRISPR-mediated bacterial immunity and advancing the development of genome editing technologies. Therefore, it is essential to develop comprehensive methods for characterizing the Cas proteins and Pro-CRISPR factors with a growing diversity. In this protocol, we provide a method encompassing protein purification, biochemical characterization, validation of protein-protein interactions, and preliminary *in vivo* functional assays in bacteria for Cas nuclease and its associated Pro-CRISPR factor. We hope this protocol will not only assist in the characterization of the CRISPR-Cas system, but also provide valuable guidance for the characterization of other nucleases or nucleic acid modification systems.

## INTRODUCTION

Since the establishment of the central dogma of molecular biology, which identifies DNA as the primary determinant of biological phenotype and heredity, manipulating organisms to achieve novel traits or functions has become pivotal for advancements in agriculture and medicine (Watson and Crick 1953). Long before the advent of genome editing, traditional methods such as hybridization breeding or inducing random mutations with chemical agents or UV radiation were extensively utilized to generate new phenotypes, albeit with limited precision and predictability (Auerbach and Robson 1944, 1946; Muller 1927). With the discovery of homing endonucleases and the development of modular genome editing tools like ZFNs and TALENs, biological technologies have entered a new era, moving from random mutation introduction to precise genome editing based on protein-guided DNA recognition and cleavage (Belfort and Roberts 1997; Bibikova *et al.* 2002; Jasin 1996; Joung and Sander 2013; Wood *et al.* 2011). However, these tools still required the design of specific DNA recognition modules based on protein for different sequences, which limited their practical applications. Therefore, the development of simple, efficient, and programmable genome editing tools became urgent.

Back in 1987, a mysterious repeat cluster, interspaced by different short sequences, was first identified in bacteria with an unknown function and later designated as clustered regularly interspaced short palindromic repeats (CRISPR) (Ishino *et al.* 1987). Further research revealed that some proteins are associated with CRISPR and were referred to as CRISPR-associated proteins (Cas) (Jansen *et al.* 2002). Almost 20 years later, the bacterial CRISPR-Cas systems were proven to function as adaptive immune mechanisms capable of recognizing and targeting nucleic acids associated with phages and other mobile genetic elements (Barrangou *et al.* 2007; Nussenzweig and Marraffini, 2020). Subsequent studies demonstrated that the Cas9 protein and two RNA components, the CRISPR RNA (crRNA) and *trans*-activating crRNA (tracrRNA), are essential for the cleavage of invading phage genomes (Deltcheva *et al.* 2011; Sapranauskas *et al.* 2011). In 2012, biochemical studies demonstrated that Cas9 could introduce dsDNA break *in vitro*, with its specificity determined by a dual guide RNA sequence composed of crRNA and tracrRNA (Gasiunas *et al.* 2012; Jinek *et al.* 2012). The design of a chimeric RNA guide, which integrates crRNA and tracrRNA with a short RNA linker loop into a single guide RNA (sgRNA), was accomplished without compromising the nuclease activity of Cas9, providing a foundation for the further development of CRISPR-based genome editing technologies (Cong *et al.* 2013).

**Figure 1:**
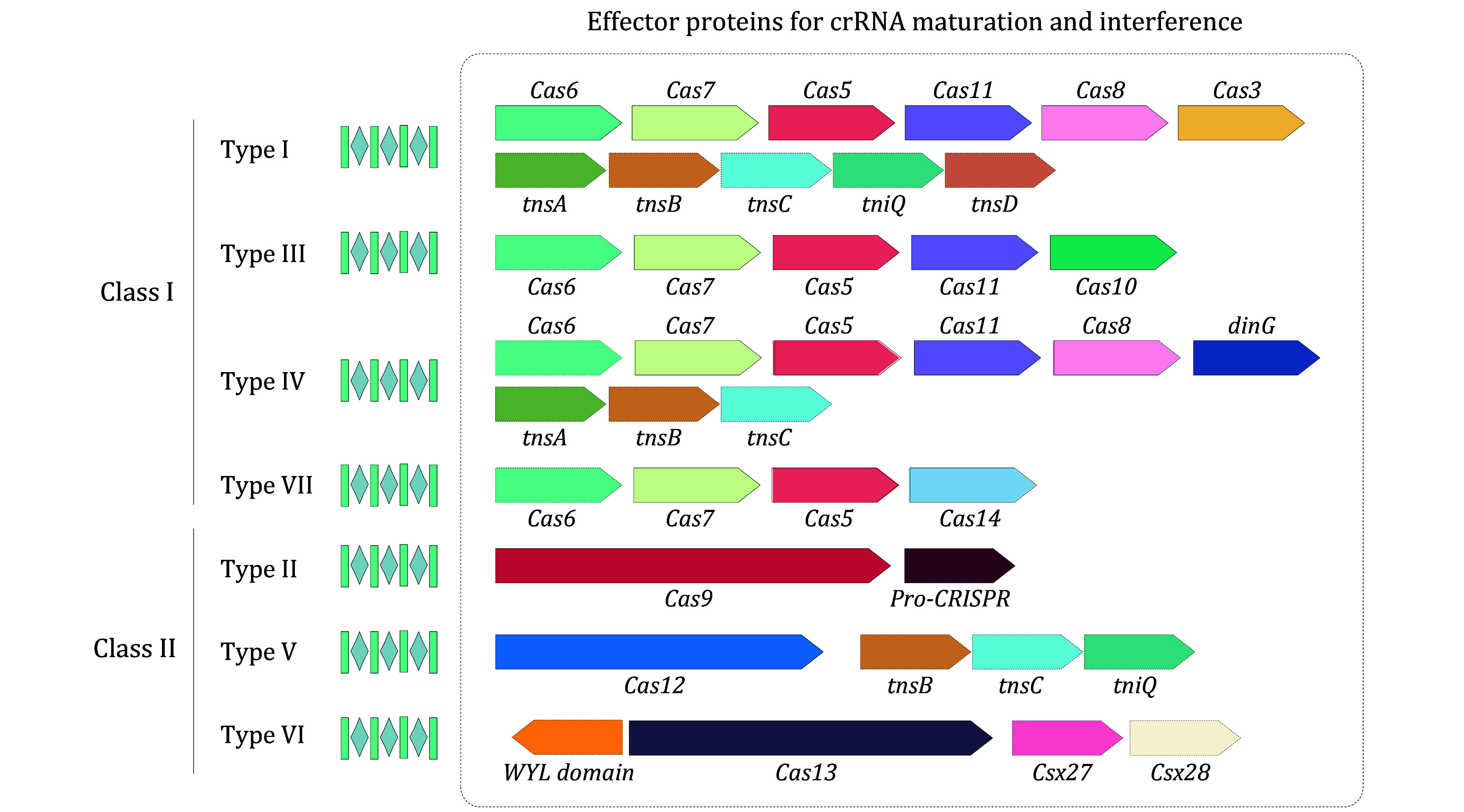
Illustration of the effector modular organizations in CRISPR-Cas systems. CRISPR-Cas systems are classified into two classes and seven types. The genes encoding proteins responsible for crRNA maturation and target interference are shown, with those found only in certain subtypes indicated by dashed outlines

According to recent research, the CRISPR-Cas system can be classified into two classes and seven types (Altae-Tran *et al.* 2023a; Makarova *et al.* 2020). Among them, Class 2 CRISPR-Cas systems are widely developed due to the unique feature of the single-nuclease effector. With the rapid advancement of genome sequencing, the diversity of Class 2 CRISPR-Cas systems has expanded, leading to the discovery of new subtypes, particularly Type V and Type IV systems, which contain 15 and 8 subtypes, respectively (Fig. 1) (Duan *et al.* 2025; Makarova *et al.* 2020; Wu *et al.* 2024; Yang and Patel 2024; Yoon *et al.* 2024). Beyond the core Cas proteins, recent studies have also uncovered additional accessory proteins, such as Tn7-like transposases, Csx27, and Csx28, which may provide new functionalities (Fig. 1) (Faure *et al.* 2019; Peters *et al.* 2017; Smargon *et al.* 2017). These accessory proteins interact with Cas proteins to form complexes or are activated by them, enhancing the defense mechanisms against phage infection or mobile genetic elements (MGE).

The Tn7-like transposons, which lack the DNA-binding module *tnsE*, were first identified in type I-F CRISPR-Cas systems without the adaptation effectors (Peters *et al.* 2017). Subsequent studies also identified the Tn7-like transposon lacking *tnsA* associated a minimal and nuclease-deficient CRISPR-Cas12k system, leading to the hypothesis that the transposition is mediated by Tn7-like transposases co-opted with Cas effectors (Faure *et al.* 2019). Consistent with the hypothesis, Tn7-like transposases can be directed to target DNA by interacting with the Cas RNP effector (Klompe *et al.* 2019; Strecker *et al.* 2019). To date, four types of CRISPR-associated transposon (CAST) systems have been identified, including types I-B, I-F, IV, and V (Rybarski *et al.* 2021). Among them, the Cas12k from the type V CAST system is the most compact effector, and it can interact with S15 and TniQ after targeting the DNA substrates, thereby recruiting TnsC and TnsB proteins for efficient RNA-guided transposition (Querques *et al.* 2021; Schmitz *et al.* 2022). Although the natural biological function of CAST systems remains unclear, some analyses indicate that their cargo genes include various anti-MGE and anti-phage systems (Benler *et al.* 2021). These unique features suggest that the CAST system may target plasmids and facilitate horizontal transfer between microbes, spreading cargo genes that contain beneficial traits, helping bacterial hosts adapt to diverse environments and defend against potential phage invasions (Benler *et al.* 2021). In addition to accessory cofactors that directly interact with Cas proteins, accessory genes, which may regulate Cas protein activity or be activated by Cas effectors to enhance CRISPR-mediated immunity, have been identified, such as *Csx27*, *Csx28*, and the *WYL-domain* in type VI-B1, VI-B2, and VI-D Cas13 systems, respectively (Smargon *et al.* 2017; Yan *et al.* 2018). Among them, the functions of the Csx28 protein, which contains both a TM domain and a HEPN domain, are the most well-studied. Studies showed that Csx28 forms an octameric, positively charged membrane-pore structure, which induces membrane depolarization, thereby reducing host metabolism and limiting the energy available for phage propagation (VanderWal *et al.* 2023). Although there is no evidence that Csx28 could directly interact with the Cas13 effector, the positively-charged Csx28 can still bind short ssRNA with high affinity, indicating an activation mechanism based on *trans*-cleavage by the Cas13 effector. Among all CRISPR systems, the Cas9 system is the most widely used due to its high efficiency in genome editing (Pacesa *et al.* 2024). Recent studies have also revealed novel accessory genes, such as Pro-CRISPR factors, within the genomic locus of type II-C CRISPR-Cas9 systems (Altae-Tran *et al.* 2023a; Zhang *et al.* 2024). Among these, PcrIIC1, one of the identified Pro-CRISPR factors, forms a heterotetrameric complex with CbCas9 and enhances its DNA binding and cleavage activity both *in vitro* and *in vivo*, paving the way for new biotechnology applications (Zhang *et al.* 2024). Further exploration of the functional diversity observed among novel-associated genes (NAGs) within CRISPR-Cas9 systems may enhance our understanding of prokaryotic adaptive immunity and expand the utility of CRISPR systems across various applications (Fig. 1).

Given the continuous discovery of novel CRISPR-Cas systems and Pro-CRISPR factors, the development of comprehensive characterization methods for these systems has become essential. This protocol outlines a method for characterizing Cas proteins, Pro-CRISPR factors, and their complexes, including protein purification, protein−protein interaction analysis, biochemical characterization, and preliminary *in vivo* functional validation. It provides valuable insights into the natural roles of Cas and Pro-CRISPR proteins, supporting the development of advanced genome-editing tools and enhancing our understanding of CRISPR systems.

## PROTOCOL OVERVIEW

**Figure 2:**
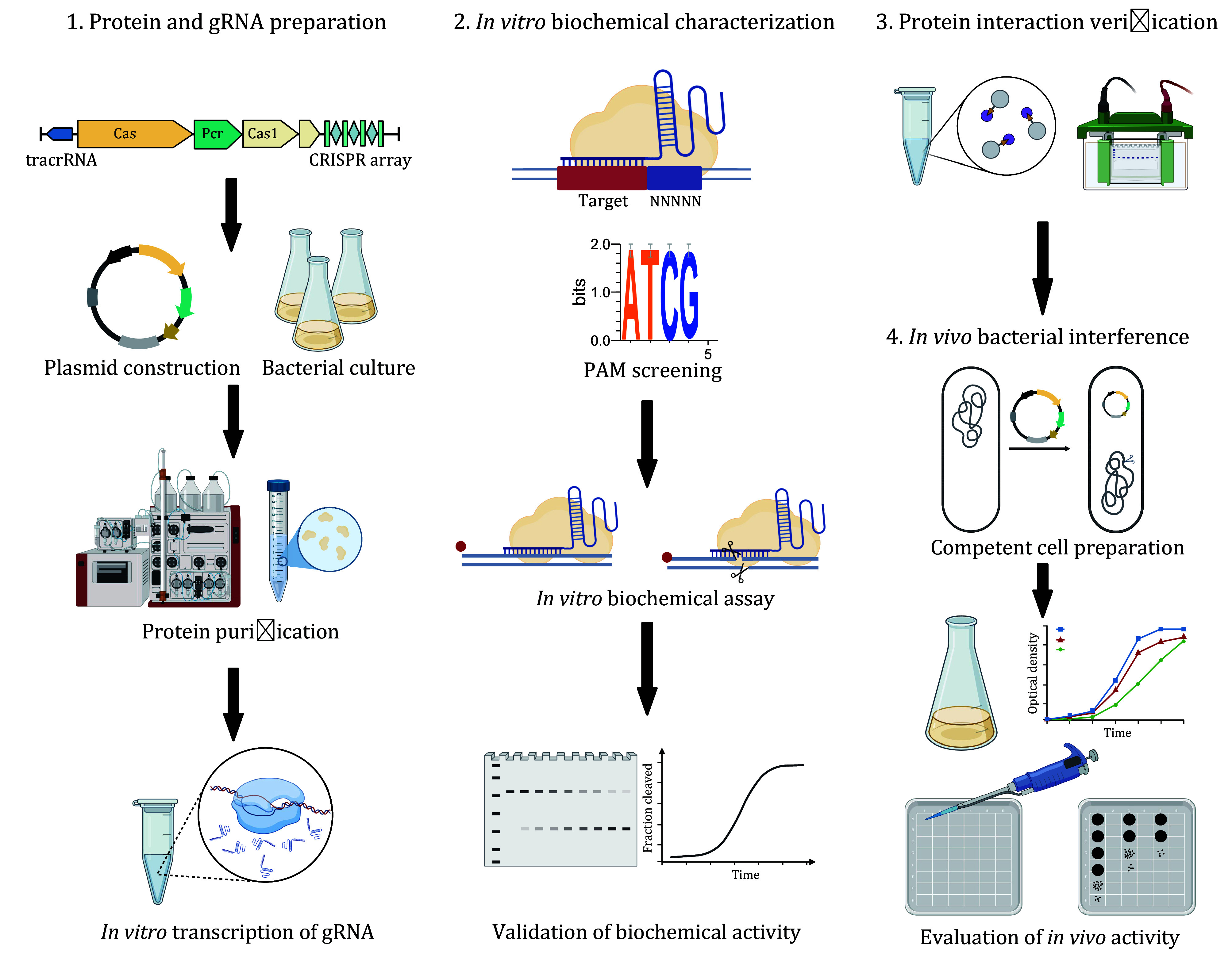
Workflow for the comprehensive characterization of novel Cas and Pro-CRISPR proteins. The experimental procedure is divided into four key steps, including protein and gRNA preparation, *in vitro* biochemical characterization, protein interaction verification, and *in vivo* bacterial interference assay, as illustrated in the figure

Based on our previous study, the entire protocol outlines the preparation of materials and equipment, reagent setup, and experimental procedures (Sun *et al.* 2023; Zhang *et al.* 2024). The procedure demonstrated a detailed, comprehensive, and step-by-step method for protein purification, gRNA preparation, *in vitro* biochemical characterization, protein interaction verification and *in vivo* bacterial interference of Cas nucleases and Pro-CRISPR factors, offering deep insights into their functional roles within CRISPR-Cas systems (Fig. 2).

## MATERIALS AND EQUIPMENT

### Materials and reagents

• *E. coli* DH5α (Sangon Biotech, Cat. #B528413-0100)

• *E. coli* BL21 (DE3) (Sangon Biotech, Cat. #B528414-0100)

• *E. coli* BW25141 (Laboratory of J.J.G.L, Tsinghua University)

• *E. coli* MG1655 (Laboratory of J.J.G.L, Tsinghua University)

• One Step Seamless Cloning Mix (CWBIO, Cat. #CW3034M)

• Bacterial Genome Extraction Kit (TIANGEN, Cat. #DP302-02)

• HighPure Maxi Plasmid Kit, EDTA (TIANGEN, Cat. #DP107-02)

• VAHTS DNA Clean Beads (Vazyme, Cat. #N411-03)

• Rapid DNA Ligation Kit (Beyotime, Cat. #D7002)

• TIANSeq Fast DNA Library Kit (Illumina) (TIANGEN, Cat. #NG102-02)

• HiPure Gel Pure DNA Kit (MGBio, Cat. #D2110-02)

• DNA Clean & Concentrator-25 (Zymo Research, Cat. #D4034)

• TSB (Solarbio, Cat. #LA0110)

• SOC sterile liquid medium (Sangon Biotech, Cat. #B540119-0100)

• IPTG (Sangon Biotech, Cat. #A600168-0100)

• Imidazole (Sangon Biotech, Cat. #A600277-0500)

• Nuclease-Free Water (Invitrogen, Cat. #AM9932)

• NTP mix (Laboratory of J.J.G.L)

• dNTP Mix (Sangon Biotech, Cat. #B500056-0005)

• T7 RNA polymerase (Laboratory of J.J.G.L, Tsinghua University)

• Murine RNase inhibitor (Vazyme, Cat. #R301-03)

• RQ1 RNase-Free DNase (Promega, Cat. #M6101)

• Heparin sodium salt (Solarbio, Cat. #H8060-1)

• Ultra GelRed (10,000×) (Vazyme, Cat. #GR501-02)

• T4 DNA polymerase (ThermoFisher Scientific, Cat. #EP0062)

• DreamTaq DNA Polymerase (ThermoFisher Scientific, Cat. #EP0702)

• Glycerol (Sangon Biotech, Cat. #A100854-0500)

• D-Glucose (Sangon Biotech, Cat. #A501991-0500)

• L-(+)-Arabinose (Sangon Biotech, Cat. #A610071-0100)

• Ni-NTA Agarose (QIAGEN, Cat. #30250)

• Ulp1 protease (Laboratory of J.J.G.L, Tsinghua University)

• TEV protease (Laboratory of J.J.G.L, Tsinghua University)

• Gel Loading Dye Purple (NEW ENGLAND Biolabs, Cat. #B7024S)

• Tris (2-Carboxyethyl) phosphine Hydrochloride (TCEP) (GLODBIO, Cat. #GC10529)

• DL-Dithiothreitol (DTT) (Sangon Biotech, Cat. #A620058-0025)

• 4-(2-Hydroxyethyl)piperazine-1-ethane-sulfonic acid (HEPES) (Merck, Cat. #H3375-1KG)

• Phenylmethyl sulfonyl fluoride (PMSF) (Sangon Biotech, Cat. #A100754-0025)

• Magnesium chloride hexahydrate (MgCl_2_-6H_2_O) (Sangon Biotech, Cat. #A601336-0500)

• T-octylphenoxypolyethoxyethanol (Triton X-100) (Sangon Biotech, Cat. #A110694-0500)

• Spermidine (MERCK, Cat. #S2626)

• Formamide (MERCK, Cat. #F15706)

• Sodium dodecyl sulfate (SDS) (MERCK, Cat. #L3771-1KG)

• Bromophenol Blue (BPB) (MERCK, Cat. #B0126)

• Xylene cyanole FF (MERCK, Cat. #X4126)

• Ethylenedinitrilotetraacetic acid (EDTA) (MERCK, Cat. #EDS-1KG)

• Trizma base (Tris) (MERCK, Cat. #T1503-10KG)

• Urea (Sangon Biotech, Cat. #A600148-0005)

• Polysorbate 20 (Tween20) (MCE, Cat. #9005-64-5)

• 5-mL HiTrap Heparin HP affinity column (GE Healthcare, Cat. #17040703)

• 5-mL MBPTrap HP column (GE Healthcare, Cat. #28918780)

• 5-mL HiTrap Q HP anion exchange chromatography column (GE Healthcare, Cat. #17115401)

• 30-kDa molecular weight cut-off centrifugal filters (Merck, Cat. #UFC903096)

• 10-kDa molecular weight cut-off centrifugal filters (Merck, Cat. #UFC901024)

• 3-kDa molecular weight cut-off centrifugal filters (Merck, Cat. #UFC900324)

• Strep-Tactin XT 4Flow resin (IBA Lifesciences, Cat. #2-5010-002)

• RED-tris-NTA 2nd Generation dye(NanoTemper technologies, Cat. #MO-L018)

• Coomassie Brilliant Blue R-250 (Sangon Biotech, Cat. #A100472-0025)

• Superdex 200 Increase 10/300 gel filtration column (GE Healthcare, Cat. #28990944)

• Superdex 200 Increase 3.2/300 (GE Healthcare, Cat. #28990946)

#### • Superose 6 Increase 5/150 GL gel filtration column (GE Healthcare, Cat. #29091597)

### Equipment

• Biowave Cell Density Meter CO8000 (VWR, Cat. #490005-908)

• JY99-IIDN Ultrasonic Homogenizer (SCIENTZ)

• AKTA Pure 25M (Cytiva, Cat. #29018226)

• AKTA Micro (Cytiva, Cat. #29302479)

• JY-SCZ8 Vertical Electrophoresis Tank (JUNYI Electrophoresis Co, Cat. #JY-SCZ8)

• JY02 UV Transilluminator (JUNYI Electrophoresis Co, Cat. #JY02)

• NanoDrop One (ThermoFisher Scientific, Cat. #840-317400)

• Amersham Typhoon 5 (Cytiva, Cat. #29187191)

• Bio-Rad Mini-PROTEAN Tetra Vertical Electrophoresis System (BIO-RAD, Cat. #1658001)

• NanoTemper Monolith (NanoTemper technologies)

• Eppendorf Eporator (BIO-RAD, Cat. #4309000027)

### Software

• ImageJ software 1.54g

• GraphPad Prism Ver. 8

• NanoTemper MO. affinity analysis software

## BUFFER SOLUTION

• Lysis buffer (20 mmol/L HEPES-Na pH 7.5, 800 mmol/L NaCl, 20 mmol/L imidazole, 10% glycerol, 1 mmol/L TCEP, and 1 mmol/L PMSF)

• Wash buffer (20 mmol/L HEPES-Na pH 7.5, 800 mmol/L NaCl, 40 mmol/L imidazole, 10% glycerol, 1 mmol/L TCEP, and 1 mmol/L PMSF)

• Dialysis buffer (20 mmol/L HEPES-Na pH 7.5, 400 mmol/L NaCl, 10% glycerol, 1 mmol/L TCEP, and 1 mmol/L PMSF)

• Heparin buffer A (20 mmol/L HEPES-Na pH 7.5, 300 mmol/L NaCl, 10% glycerol, and 3 mmol/L DTT)

• Heparin buffer B (20 mmol/L HEPES-Na pH 7.5, 2 mol/L NaCl, 10% glycerol, and 3 mmol/L DTT)

• S200 buffer (20 mmol/L HEPES-Na pH 7.5, 200 mmol/L NaCl, 10% glycerol, 2 mmol/L DTT)

• IVT buffer (30 mmol/L Tris-HCl pH 8.1, 25 mmol/L MgCl_2_, 0.01% Triton, 2 mmol/L spermidine)

• 2× formamide loading (95% formamide, 0.02% SDS, 0.02% BPB, 0.01% xylene cyanole FF, 1 mmol/L EDTA)

• Assembly buffer (150 mmol/L NaCl, 20 mmol/L HEPES-Na pH 7.5, 1 mmol/L TCEP, 5 mmol/L MgCl_2_, and 1% glycerol)

• Cleavage buffer (20 mmol/L HEPES-Na pH 7.5, 150 mmol/L NaCl, 10 mmol/L MgCl_2_, 1% Glycerol, and 1 mmol/L TCEP)

• 2× Urea loading (8 mol/L urea, 2 mmol/L Tris-HCl pH 7.5)

• MST buffer (20 mmol/L HEPES-Na pH 7.5, 150 mmol/L NaCl, 5 mmol/L MgCl_2_, 2 mmol/L DTT and 0.05%Tween20)

• Reconstitution buffer (20 mmol/L HEPES-Na pH 7.5, 150 mmol/L NaCl, 5 mmol/L MgCl_2_, 0.5% glycerol, and 1 mmol/L TCEP)

• Pull-down buffer (20 mmol/L HEPES-Na pH 7.5, 150 mmol/L NaCl, 5 mmol/L MgCl_2_, 1% glycerol, 1 mmol/L TCEP, and 0.05% Tween20)

## PROCEDURE

### Pre-step analysis

**Figure 3:**
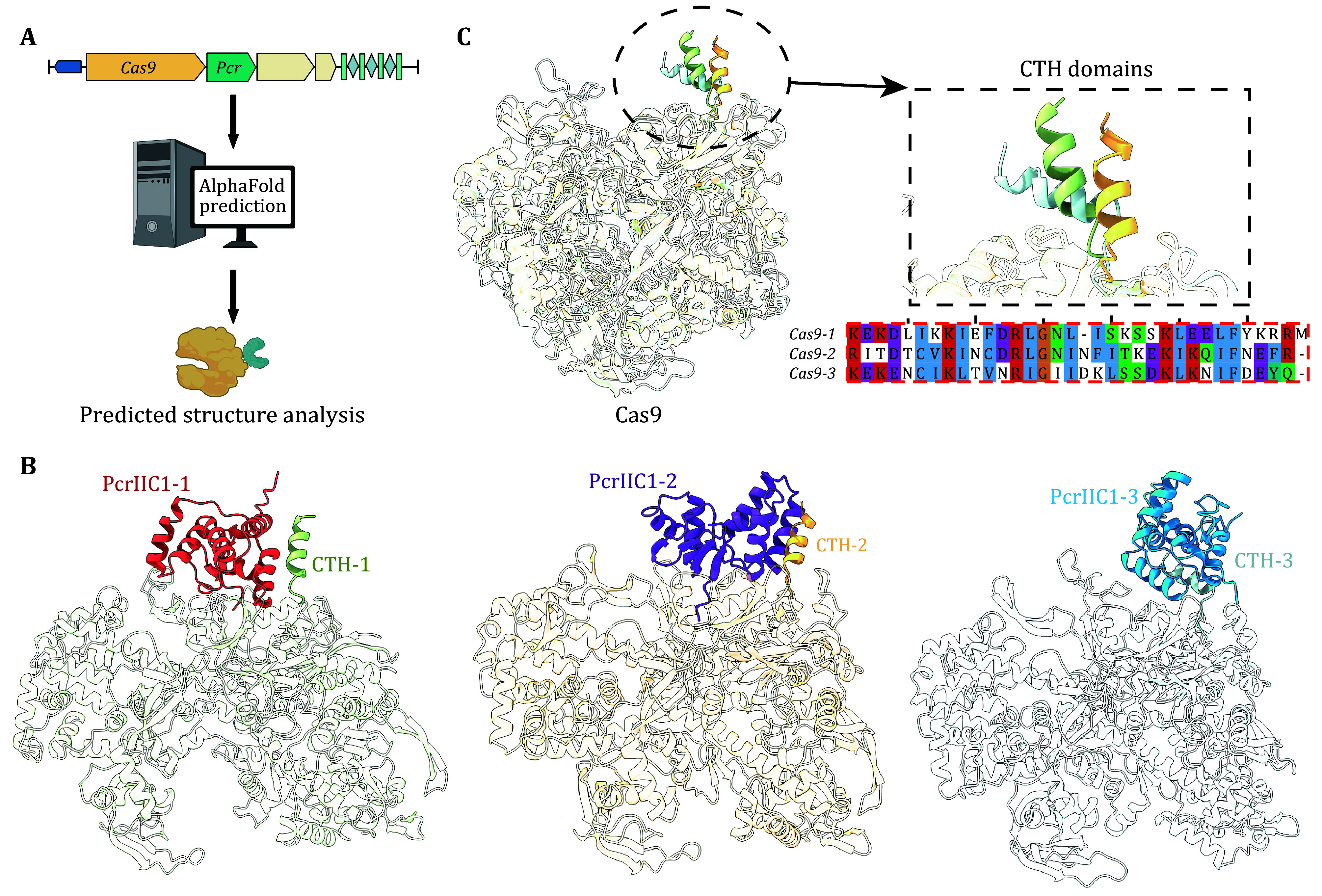
Protein interaction validation before experiments. **A** Model depicting analysis of protein interaction validation. **B** The predicted structures of three different Cas9-PcrIIC1 complexes. All PcrIIC1 proteins interact with the CTH domain of Cas9s. **C** Structure alignment of three PcrIIC1-associated Cas9s (left), and local structural model and primary sequence alignment of CTH domains (right)

Before the biochemical characterization of Cas proteins and Pro-CRISPR factors, we recommend using AlphaFold2-Multimer or AlphaFold3 to predict the protein structures and interactions between selected Cas proteins and Pro-CRISPR (Abramson *et al.* 2024; Jumper *et al.* 2021). Subsequently, using ChimeraX or PyMOL to visualize the predicted results followed by structural alignment to identify shared features among different complexes. Here, we used PcrIIC1-associated Cas9 systems as an example. Structure predictions revealed that all PcrIIC1 proteins interact with the C-terminus helix (CTH domain) of their corresponding Cas9 proteins. Sequence and structure alignments of Cas9 proteins further confirmed that the CTH domain is highly conserved in both primary sequence and three-dimensional structure among Cas9 proteins associated with PcrIIC1, supporting the hypothesis of their interactions (Fig. 3).

### Step 1: Protein and gRNA preparation

#### Step 1.1: Plasmid construction [TIMING 2−3 d]

Step 1.1.1: The genes encoding Cas and Pro-CRISPR proteins can be amplified from the native host genome or synthesized with a codon optimized for expression in *E. coli*. For protein expression, clone the *Cas* gene into a pET28a-based vector with an N-terminal 10× His-SUMO tag, while inserting the *Pro-CRISPR* gene into a pET28a-based vector with an N-terminal 6× His-MBP tag via Gibson assembly using One Step Seamless Cloning Mix.

**[OPTIONAL]** Cultivation of native host bacteria. Remove the bacterial strain from the −80°C freezer and streak it onto a TSB agar plate. Incubate the plate at 30°C overnight. Pick a single colony the next day, inoculate it into 5 mL of TSB liquid medium, and culture the bacteria overnight at 30°C with shaking at 220 r/min. Subsequently, centrifuge the bacterial culture at 3500 r/min for 10 min at room temperature and extract host genomic DNA using the Bacterial Genome Extraction Kit, following the manufacturer’s instructions.

Step 1.1.2: For the construction of protein mutants. Amplify the target gene with primers containing the desired mutations and further construct the plasmid via Gibson assembly using One Step Seamless Cloning Mix.

#### Step 1.2: Protein purification [TIMING 3−5 d]

Step 1.2.1: Transform the protein expression plasmids into *E. coli* BL21 (DE3) and culture the bacteria overnight on LB agar plates containing the appropriate antibiotic at 37°C.

Step 1.2.2: Next day, pick a single colony from the plate and inoculate it into 100 mL of LB medium with the same antibiotic. Incubate the culture at 37°C with shaking at 200 r/min as seed.

**[CRITICAL STEP]** Perform colony PCR before picking a single colony to verify the presence of the plasmid.

Step 1.2.3: Transfer 10 mL seed culture into 1 L fresh LB medium containing the appropriate antibiotic and incubate the bacteria at 37°C with shaking at 200 r/min until the optical density at 600 nm (OD600) reaches 0.8−1.2.

Step 1.2.4: Induce protein expression by adding IPTG with a final concentration of 0.22 mmol/L and further incubate the culture at 16°C for 18−20 h.

**[CRITICAL STEP]** Cool down the temperature of the bacterial culture to 16°C for 1−2 h before adding IPTG.

Step 1.2.5: Harvest the bacterial cells by centrifugation at 3500 r/min for 10 min at 4°C, and resuspend the pellet from the 1 L culture in 30 mL of Lysis buffer.

Step 1.2.6: Lyse the cells by sonication and centrifuge the lysate at 13,000 r/min for 80 min at 4°C.

Step 1.2.7: Collect and apply the supernatant to a gravity column containing Ni-NTA resin pre-equilibrated with Wash buffer. Allow the mixture to flow through the column two to three times to ensure that the majority of the protein binds to the resin.

Step 1.2.8: Wash the column with 10 column volumes (CV) of Wash buffer supplemented with an imidazole gradient from 40 to 60 mmol/L to remove any nonspecifically bound proteins.

##### Step 1.2.9: Further purification of Cas proteins

Step 1.2.9.1: Resuspend the resin with 5 CV of Wash buffer supplemented with Ulp1 protease at 4°C for 1 h to remove the N-terminal 10× His-SUMO tag.

Step 1.2.9.2: After digestion with Ulp1 protease for Cas, dilute the salt concentration of the reaction mixture to 400−500 mmol/L NaCl. Load the mixture onto a 5-mL HiTrap Heparin HP column and elute the protein with a linear gradient of Heparin buffer A to buffer B. Collect each fraction and analyze them by SDS-PAGE.

**[CRITICAL STEP]** Ensure that the salt concentration of the Cas protein sample is reduced before loading it onto the Heparin HP column.

Step 1.2.9.3: Pool the fractions containing the protein. Concentrate the pooled fractions to approximately 0.5 mL using a 30-kDa molecular weight cut-off centrifugal filter.

Step 1.2.9.4: Load the concentrated sample onto a Superdex 200 Increase 10/300 gel filtration column equilibrated with S200 buffer.

##### Step 1.2.10: Further purification of Pro-CRISPR factors

Step 1.2.10.1: Elute the bound protein from the Ni-NTA resin with Wash buffer supplemented with 200 mmol/L imidazole.

Step 1.2.10.2: Collect the elution fractions, add TEV protease to cleave the N-terminal 6× His-MBP tag, and dialyze the mixture overnight at 4°C in the Dialysis buffer.

Step 1.2.10.3: After digestion with TEV protease for Pro-CRISPR protein, dilute the salt concentration of the reaction mixture to 300 mmol/L NaCl. Apply the sample to a 5-mL MBPTrap HP column and a 5-mL HiTrap Q HP column connected in tandem to remove the 6× His-MBP tag and extra nucleic acid contaminations.

Step 1.2.10.4: Concentrate the flow through using 3-kDa molecular weight cut-off centrifugal filters to 0.5 mL and load the sample onto a Superdex 200 Increase 10/300 gel filtration column equilibrated with S200 buffer.

Step 1.2.11: Analyze each fraction by SDS-PAGE. Concentrate the purified protein and store it at −80°C after flash-frozen in liquid nitrogen.

#### Step 1.3: In vitro transcription of gRNA [TIMING 2 d]

Step 1.3.1: Prepare and amplify the DNA template for IVT using a forward primer containing the T7 promoter sequence and several reverse primers via overlap PCR.

Step 1.3.2: Further amplify the final DNA template for IVT using the preliminary PCR product as the amplification template (1 μL for a 50 μL PCR reaction), with two primers located at the 5’ and 3’ ends of the target DNA sequence.

Step 1.3.3: Add 50 μL final PCR products into 750 μL IVT buffer supplemented with 4 mmol/L NTP mix and 0.4 mg/mL T7 RNA polymerase, 1000 units RNase Inhibitor, and incubate at 37°C for 8 h.

**[CRITICAL STEP]** If RNA degradation or impurities are generated, consider lowering the T7 RNA polymerase concentration from 0.4 to 0.04 mg/mL and reduce the incubation time to 4−8 h to improve purity.

Step 1.3.4: Add 25 units of RNase-free DNase I to the IVT reaction and incubate the mixture at 37°C for 30 min to remove the DNA templates.

Step 1.3.5: After DNA digestion, centrifuge the sample at 12,000 r/min, 4°C for 5 min to remove the precipitates.

Step 1.3.6: Mix the supernatant with 2× formamide loading and load the mixture into 10% Urea-PAGE for electrophoresis in SCZ-8 Vertical Electrophoresis Tank, with parameters set to 45 W for 2 h.

Step 1.3.7: View and excise the gel region containing the target RNA band using the JY02 UV Transilluminator. Crush the gel slice and soak it in soaking buffer for 12 h at 4°C in a 50-mL centrifuge tube.

Step 1.3.8: The next day, centrifuge the sample at 3500 r/min at 4°C for 15 min. Filter the supernatant through a 0.22 μm filter to obtain the dissolved gRNA solution.

Step 1.3.9: Concentrate the dissolved gRNA using 10-kDa molecular weight cut-off centrifugal filters. Measure the RNA concentration using NanoDrop One, and store the gRNA at −80°C after flash-frozen in liquid nitrogen.

### Step 2: *In vitro* biochemical characterization of Cas effectors

#### Step 2.1: PAM screening assay [TIMING 2−3 d]

Step 2.1.1: Clone the synthesized DNA fragment containing randomized nucleotides and the target sequence into the pUC19 vector using One Step Seamless Cloning Mix.

Step 2.1.2: Transform the pUC19 plasmid containing the PAM library into *E. coli* DH5α and incubate the bacteria overnight at 37°C on LB agar plates (100 μg/mL Ampicillin).

Step 2.1.3: Harvest and culture all colonies in 100 mL LB medium (100 μg/mL Ampicillin) at 37°C for 4 h. Then, extract the plasmids using the HighPure Maxi Plasmid Kit.

**[CRITICAL STEP]** The randomized nucleotide sequence should be verified by Sanger sequencing or NGS before the PAM screening assay to ensure that the fragment is correctly inserted into the plasmid.

Step 2.1.4: Incubate 1 μmol/L Cas protein with 1.2 μmol/L sgRNA in Assembly buffer at room temperature (RT) for 30 min to form active RNP complexes.

Step 2.1.5: Add plasmid DNA containing the PAM library with a final concentration of 20 nmol/L to the assembled RNP complexes in Cleavage buffer. Incubate the reaction mixture at 37°C for 1 h to allow DNA cleavage.

Step 2.1.6: Quench the reaction by adding Gel Loading Dye Purple supplemented with 20 mmol/L EDTA and 50 μg/mL heparin sodium salt.

Step 2.1.7: Analyze the cleaved products by electrophoresis on a 1.2% agarose gel pre-stained with Ultra GelRed to visualize the DNA fragments.

Step 2.1.8: Purify the products using VAHTS DNA Clean Beads, following the manufacturer’s instructions, and dilute the purified DNA to 40 μL.

Step 2.1.9: Add 1 μL T4 DNA polymerase and 1 μL 10 mmol/L of dNTP Mix to the DNA sample in a total volume of 50 μL reaction for the end repair of the linearized DNA. Incubate the reaction at 11°C for 20 min, followed by enzyme inactivation at 75°C for 10 min.

Step 2.1.10: Incubate end-repair sample with 1 μL DreamTaq DNA Polymerase and 1 μL 10 mmol/L of dATP at 72°C for 30 min to add dA tails to the 3’ ends of the DNA. Then, purify the reaction products using VAHTS DNA Clean Beads and dilute the purified DNA with 20 μL of Nuclease-Free water.

Step 2.1.11: Add a synthesized dsDNA fragment containing 3’ dT overhangs to the 20 μL dA-tailed DNA sample at a final concentration of 1 μmol/L, and ligate them using the Rapid DNA Ligation Kit in a total reaction volume of 50 μL. Then, purify the reaction products using VAHTS DNA Clean Beads and dilute the purified DNA with 25 μL of Nuclease-Free water.

Step 2.1.12: Amplify the DNA fragments containing the recognized PAM sequence using a primer that binds to the synthesized dsDNA and another primer that binds to the 120-bp upstream region of the PAM, with the ligated DNA as the template, and further purify the PCR products using VAHTS DNA Clean Beads.

Step 2.1.13: Prepare the NGS library for Illumina Novaseq PE150 sequencing using the TIANSeq Fast DNA Library Kit (Illumina), following the manufacturer’s protocol.

Step 2.1.14: Process the raw data in FASTQ format from DNA sequencing using Trim Galore to remove adapters and low-quality reads. Assess the quality of cleaned data by calculating the Q20, Q30, and GC content using FastQC.

Step 2.1.15: Merge the PE150 reads to longer reads by FLASH and align the results to the sequence of the PAM library. Generate the PAM preference logo using WebLogo.

**Figure 4:**
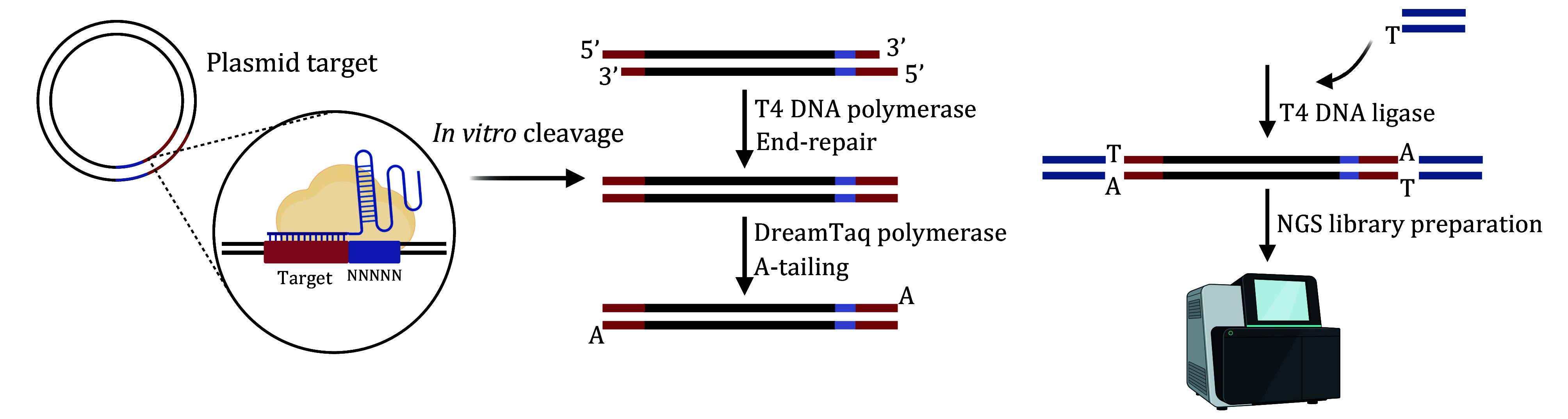
Illustration demonstrating the detailed protocol for PAM screening assay, including plasmid cleavage, end-repair, A-tailing, DNA ligation, and NGS library preparation

**[TIP]** The detailed protocol for the PAM screening assay is displayed in Fig. 4.

#### Step 2.2: DNA cleavage assay [TIMING 2−4 h]

Step 2.2.1: Prepare the target dsDNA substrate by PCR extension using a longer ssDNA template and a 5’-Cy5-labeled 16-nt primer complementary to the 3’-end of the template. Purify the DNA target substrate using a Clean & Concentrator-25 kit.

**[CRITICAL STEP]** When preparing the dsDNA substrates via PCR amplification, adjust the extension time based on the length of the longer ssDNA template to avoid the generation of unwanted products.

Step 2.2.2: For each cleavage reaction, prepare the RNP complex by mixing the Cas protein (0.1−1 μmol/L) with the sgRNA (0.12−1.2 μmol/L) in the assembly buffer. Incubate the mixture at RT for 30 min to allow the formation of the active RNP complex.

Step 2.2.3: Mix the prepared dsDNA substrate (final concentration of 20 nmol/L) with the RNP complex. Incubate the reaction at 37°C for 1 h in Cleavage buffer to allow DNA cleavage.

Step 2.2.4: After incubation, collect and quench the samples at different time points with 2× Urea loading, supplemented with 20 mmol/L EDTA and 50 μg/mL heparin sodium salt.

Step 2.2.5: Analyze the cleavage products by electrophoresis on a 15% urea-PAGE gel in SCZ-8 Vertical Electrophoresis Tank, with parameters set to 50 W for 45 min. Visualize the substrate and cleaved products using Amersham Typhoon 5 and quantify the data using ImageJ software.

Step 2.2.6: Calculate the cleavage efficiency by quantifying the percentage of cleaved products relative to the total substrate. Plot the data using the One-Phase Decay model in GraphPad Prism 8 to obtain the plateau value (maximum cleavage efficiency) and the rate constant (*k* value) for each reaction.

#### Step 2.3: Electromobility shift assay (EMSA) [TIMING 2−4 h]

Step 2.3.1: Prepare deactivated Cas-RNP effectors (at a final concentration of 8.192 μmol/L) by mixing the Cas protein and sgRNA at a molar ratio of 1:1.2 in Assembly buffer and incubate the sample at RT for 30 min to allow RNP complex formation.

**[CRITICAL STEP]** To avoid cleavage of the substrates, deactivated Cas protein mutants should be used.

Step 2.3.2: Dilute the prepared RNP complex into a series of concentrations (*e*.*g*., 0, 4, 8, 16, 32, 64, 128, 256 and 512 nmol/L, and 1.024, 2.048 and 4.096 μmol/L) using Assembly buffer. Then, add the Cy5-labeled dsDNA targets containing different PAM sequences at a final concentration of 8 nmol/L and incubate the reaction at 37°C for 30 min.

Step 2.3.3: Resolve the reaction samples on a 4% native PAGE gel at 4°C in a Bio-Rad Mini-Protean Tetra Vertical Electrophoresis System. Visualize the substrate (unbound target) and product (bound target) bands using Amersham Typhoon 5 and quantify the bands using ImageJ software.

Step 2.3.4: Plot the curve of the bound fraction by fitting the data using the [Agonist] vs. Response-Variable slope (four parameters) equation in GraphPad Prism. Calculate the equilibrium dissociation constant (*K*_D_) using the One-site Specific binding model.

#### Step 2.4: Trans-cleavage activity [TIMING 2−4 h]

**Figure 5:**
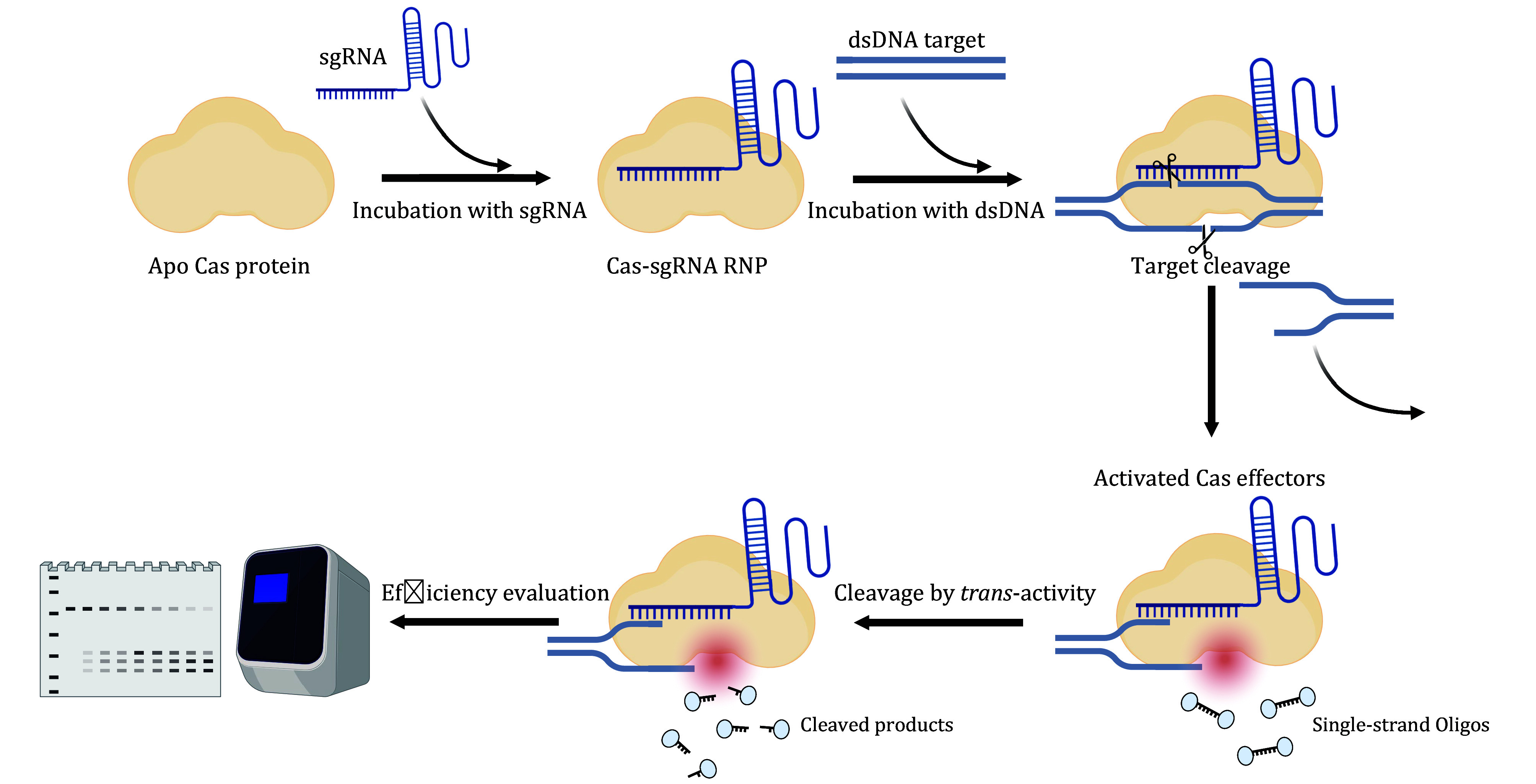
Model illustrating the process of *trans*-cleavage activation after *cis*-cleavage by Cas effectors

Some Cas variants, such as Cas12a and Cas13, not only exhibit target substrates cleavage activity but also possess *trans*-cleavage activity, degrading random ssDNA or ssRNA upon activation by target DNA binding and cleavage, which has been harnessed in nucleic acid diagnostics (Fig. 5) (Chen *et al.* 2018; Gootenberg *et al.* 2018).

Step 2.4.1: Prepare the *trans*-cleavage effector by incubating 1 μmol/L active Cas RNP with 1.5 μmol/L target dsDNA or ssDNA substrate activators in Cleavage buffer at 37°C for 30 min to allow the formation of the effector complex.

Step 2.4.2: Add 5’-Cy5-labeled random 60-nt ssDNA target of a final concentration of 20 nmol/L into the mixture and incubate the reaction at 37°C.

Step 2.4.3: After incubation, collect and quench the samples at different time points with 2× Urea loading, supplemented with 20 mmol/L EDTA and 50 μg/mL heparin sodium salt.

Step 2.4.4: Analyze the *trans*-cleavage efficiency following the procedure described in Step 2.2.

### Step 3: Protein interaction verification

#### Step 3.1: Pull-down assay [TIMING 4−6 h]

Step 3.1.1: Clone the *Pro-CRISPR (Pcr)* gene into a pET28a-based vector with an N-terminal 6× His-MBP-StreptagII protein purification tag and purify the protein as described in Step 1.

Step 3.1.2: Incubate 4 μmol/L of N-terminal StreptagII-Pcr protein with 4 μmol/L of Cas proteins in 50 μL of Reconstitution buffer at 4°C for 2 h to allow complex formation.

Step 3.1.3: Pre-wash 10 μL Strep-Tactin resins with 200 μL of Pull-down buffer three times and equilibrate the beads in 50 μL of Pull-down buffer.

Step 3.1.4: Mix the protein complex with the equilibrated Strep-Tactin resins and incubate them at 4°C for 30 min on a rotating wheel to allow binding of StreptagII-PcrIIC1 to the beads.

Step 3.1.5: Centrifuge the incubated beads at 800 r/min for 1 min, discard the supernatant, and wash the beads three times with 50 CV of Pull-down buffer to remove unbound proteins.

Step 3.1.6: Resuspend the beads in 3 CV of Pull-down buffers, incubate the bead suspension at 95°C for 10 min, centrifuge the suspension at 12,000 r/min for 2 min, and collect the supernatant.

Step 3.1.7: Analyze the supernatant by SDS-PAGE and stain with Coomassie Brilliant Blue R-250 to visualize the proteins.

#### Step 3.2: Microscale thermophoresis [TIMING 3−4 h]

Step 3.2.1: Prepare the N-terminal 10× His-tagged deactivated Cas protein as described in Step 1.

Step 3.2.2: Incubate 5 μmol/L His-tagged Cas with 6 μmol/L gRNA at RT for 30 min in MST buffer to form Cas RNP complex. Add 7.5 μmol/L dsDNA targets to the RNP complex and incubate for 30 min at 25°C in MST buffer to allow the formation of the ternary complex.

Step 3.2.3: Dilute the apo Cas protein or Cas complexes to 50 nmol/L and label them with the RED-tris-NTA 2nd Generation dye, following the manufacturer’s instructions.

Step 3.2.4: Incubate the labeled effector with a dilution series of Pcr protein at 25°C and measure the binding affinity using premium capillaries at 40% LED excitation and medium MST power.

Step 3.2.5: Analyze the data and visualize the binding curves using NanoTemper MO. affinity analysis software.

#### Step 3.3: Size exclusion chromatography assay [TIMING 3−4 h]

Step 3.3.1: Incubate Apo Cas protein with gRNA at a 1:1.2 molar ratio in Reconstitution buffer for 30 min at RT to form the Cas RNP complex.

Step 3.3.2: Add DNA target to the Cas RNP complex at a 1.5:1 molar ratio and incubate the mixture in Reconstitution buffer for 45 min at RT to assemble the Cas-gRNA-dsDNA ternary complex.

Step 3.3.3: Mix the Pcr protein with Apo Cas or Cas complexes at a 3:1 molar ratio and incubate the mixture for 2 h on ice in a Reconstitution buffer to allow the formation of Cas-Pcr effector.

Step 3.3.4: Separate all mixed samples using the SuperdexTM 200 Increase 3.2/300 or the SuperoseTM 6 Increase 5/150 GL gel filtration column in the Reconstitution buffer.

Step 3.3.5: Analyze and visualize all sample fractions using 12% SDS-PAGE.

### Step 4: Characterization of Pro-CRISPR factor

Here, we used PcrIIC1 as an example to illustrate the protocol. Given that different proteins may exhibit distinct properties, we strongly recommend thoroughly reviewing relevant literature before delving deeper into the characteristics of specific Pro-CRISPR factors. PcrIIC1 is annotated as a member of the PIN-domain superfamily, which has been reported to function as an endonuclease on ssRNA and a bacterial toxin. Therefore, the following experiments were conducted to investigate its activity and characteristics.

#### Step 4.1: In vitro cleavage assay [TIMING 2 h]

Step 4.1.1: Dilute synthesized 5’or 3’-Fam-labeled ssDNA and ssRNA substrates to a final stock concentration of 1 μmol/L. Amplify the labeled dsDNA substrate from pUC19-based plasmids using a 5’-Cy5-labeled 16-nt primer and a non-labeled primer. Purify the product using the HiPure Gel Pure DNA Kit and dilute it to 100 nmol/L.

Step 4.1.2 Initiate the cleavage reaction by adding 1 μmol/L PcrIIC1 with 20 nmol/L of the respective ssDNA, ssRNA, or dsDNA substrates in the Cleavage buffer. Incubate the mixture at 37°C.

Step 4.1.3: After incubation, collect the samples at different time points and quench the reaction with 2× Urea loading, supplemented with 20 mmol/L EDTA and 50 μg/mL heparin sodium salt.

Step 4.1.4: Analyze the cleavage efficiency of PcrIIC1 using the same protocol described in Step 2.2.

#### Step 4.2: In vivo toxicity assay [TIMING 6−7 d]

Step 4.2.1: Remove the *E. coli* BW25141 from the −80°C freezer and streak it onto an LB agar plate. Culture the bacteria at 37°C overnight.

Step 4.2.2: Pick a single colony from the plate and inoculate it into 100 mL of antibiotic-free LB medium the next day. Incubate the culture at 37°C with shaking at 200 r/min overnight as the seed culture.

Step 4.2.3: Transfer 10 mL of the overnight seed culture into 1 L of antibiotic-free LB medium and incubate it at 37°C with shaking until the OD600 reaches 0.4−0.5.

**[CRITICAL STEP]** An OD600 of 0.4−0.5 is the optimal range for preparing electrocompetent cells.

Step 4.2.4: Harvest the cells by centrifugation at 3500 r/min for 10 min at 4°C. Discard the supernatant and resuspend the pellet in 100 mL of pre-cold sterile water.

Step 4.2.5: Wash the cells three times with 800 mL of pre-cold sterile water, followed by two washes with 400 mL of pre-cold 10% sterile glycerol. Centrifuge at 3500 r/mim for 10 min at 4°C between each wash.

Step 4.2.6: After the final wash, resuspend the cells in 2.5 mL of pre-cold 10% sterile glycerol. Aliquot 100 μL of the resuspended cells into 1.5 mL EP tubes.

Step 4.2.7: Store the aliquoted cells at −80°C after flash-frozen in liquid nitrogen for long-term storage as electrocompetent cells.

Step 4.2.8: Clone *MBP*, *SUMO*, *PcrIIC1,* or *VapC* toxin gene into pBAD-driven arabinose inducible plasmids.

Step 4.2.9: Electroporate the inducible plasmids into 50 μL electrocompetent *E. coli* BW25141 cells using a 0.2 cm cuvette at 2.5 kV with an Eppendorf Eporator. Recover the cells in 5 mL SOC medium at 37°C for 1.5 h, then culture overnight at 37°C on an LB agar plate supplemented with 0.5% glucose.

Step 4.2.10: Pick a single colony from the LB agar plate and inoculate it into 10 mL of LB medium supplemented with 0.5% glucose. Incubate the culture overnight at 37°C with shaking at 200 r/min overnight.

Step 4.2.11: Centrifuge the culture at 3500 r/min for 10 min, discard the supernatant, and wash the cells three times with 10 mL of glucose-free LB medium to remove glucose from the solution. Resuspend the cells in a glucose-free LB medium and adjust the OD600 to 0.4.

Step 4.2.12: Dilute the suspended cells 100-fold and culture them in LB medium supplemented with 1% glucose to inhibit protein expression, or in LB medium supplemented with 25 mmol/L arabinose to induce protein expression. Load the culture into the Bioscreen C Microbiological Growth Analyzer, and incubate them at 37°C with shaking at 200 r/min for 22 h, while taking OD600 measurements every 2 h.

**Figure 6:**
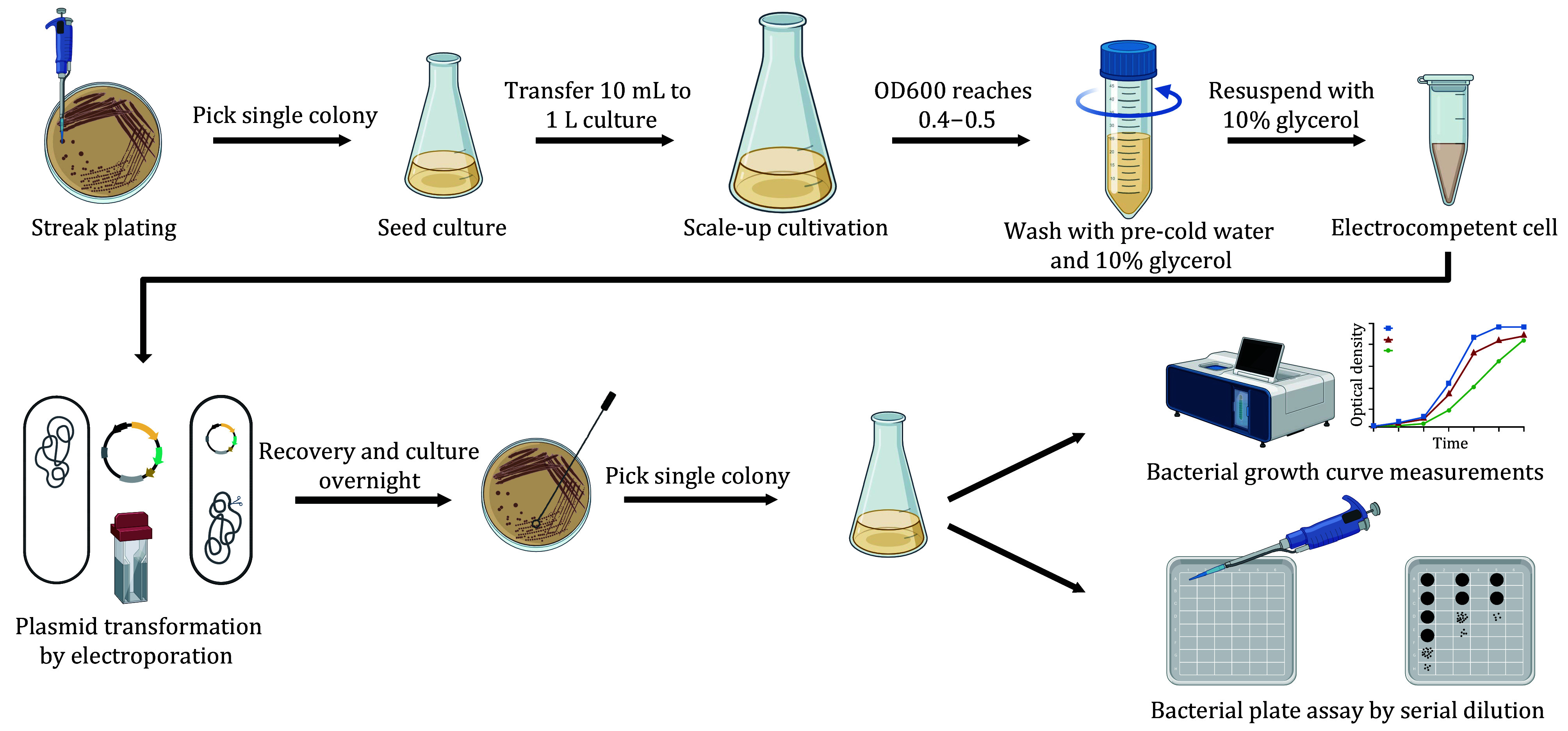
Model showing the detailed protocol for electrocompetent cell preparation, plasmid transformation, and *in vivo* functional assays for Cas and Pro-CRISPR proteins

**[TIP]** The detailed protocols for electrocompetent cell preparation and bacterial growth curve measurements are displayed in Fig. 6.

### Step 5: Function verification of Cas effector with Pro-CRISPR factor

#### Step 5.1: In vitro biochemical characterization of the complex [TIMING 4−5 h]

Step 5.1.1: Incubate Cas with sgRNA at RT for 30 min to form the RNP complex, then immediately add Pcr protein to the complex and continue incubation on ice for 2 h to allow the formation of Cas-Pcr complex.

Step 5.1.2: Perform the biochemical assays as described in Step 2 using the Cas-Pcr effector instead of the Cas effector, including PAM screening, DNA interference, EMSA, and *trans*-cleavage.

#### Step 5.2: In vivo bacterial interference assay [TIMING 5 d]

Step 5.2.1: Prepare the *E. coli* MG1655 electrocompetent cells as described in Step 4.2.

Step 5.2.2: Clone the *Cas* gene, or both *Cas* and *Pcr* genes, respectively, downstream of the IPTG-inducible Trc promoter in modified pCDF-Duet vectors, along with sgRNAs driven by the J23119 promoter, which contains two SapI sites for spacer insertion by Golden Gate assembly.

Step 5.2.3: Electroporate the IPTG-inducible plasmids into 75 μL electrocompetent *E. coli* MG1655 cells using a 0.2 cm cuvette at 2.5 kV with an Eppendorf Eporator. Recover the cells in 5 mL SOC medium at 37°C for 1.5 h.

Step 5.2.4: Transfer the recovered cells into 5 mL of LB medium supplemented with 0.2 mmol/L IPTG, and incubate the culture at 37°C for 8 h with shaking at 250 r/min to induce protein expression.

Step 5.2.5: Spot 6 µL of the culture from gradient dilutions (10^0^ to 10^−5^) onto LB agar plates. Incubate the plates at 37°C overnight to assess the bacterial genome interference efficiency.

**[TIP]** The detailed protocols for electrocompetent cell preparation and *in vivo* bacterial functional assay are shown in Fig. 6.

## ADVANTAGES AND LIMITATIONS OF THIS PROTOCOL

This detailed step-by-step protocol provides a standardized guide for the *in vitro* characterization of novel CRISPR-Cas systems with potential pro-CRISPR factors, facilitating a comprehensive analysis of various aspects of these systems. Additionally, the protocol can be further applied to nuclease systems involving non-Cas proteins, such as Argonaute proteins and restriction endonucleases, including protein purification, DNA interference, and the validation of potential accessory genes. However, this protocol primarily focuses on the *in vitro* characterization and validation, additional methods based on *in vivo* experiments are required for further characterization of the biological functions and significance of these systems. Given that more novel CRISPR-Cas systems and associated genes continue to be discovered, the development of related protocols will need to be expanded and refined.

## DISCUSSION

Due to its high efficacy and easy programmability, CRISPR-Cas-based genome editing has become a prominent biotechnology in the field of molecular biology and medicine. It has been shown that the Cas9 and Cas12 families originated from the IS200/605 transposon family, evolving through prolonged interactions between bacteria and viruses into multi-domain proteins, contributing to the diversity of identified Cas nucleases (Altae-Tran *et al.* 2021). The characterization of these ancestral RNA-guided TnpB and IscB from IS200/605 has addressed the delivery challenges posed by the large molecular size of Cas9 and Cas12a, providing more efficient genome editing tools. Furthermore, recent studies have identified a TnpB-like Fanzor protein from eukaryotic cells, which also exhibits RNA-guided nuclease activity (Saito *et al.* 2023). The discovery of eukaryotic Fanzor has significantly expanded the scope of the CRISPR-Cas system, offering deeper insights into molecular evolution and presenting promising prospects for genome editing. In addition to the discovery of RNA-guided nucleases, recent research has also highlighted the significance of accessory genes in these systems, further driving the development of genome editing applications (Altae-Tran *et al.* 2023a; Altae-Tran *et al.* 2023b; Zhang *et al.* 2024). In the prolonged evolutionary arms race between bacteria and phages, phages have evolved anti-CRISPR proteins that evade the host’s immune attack by inhibiting CRISPR through various mechanisms, contributing to more precise control of CRISPR-mediated genome editing (Maxwell 2017; Pawluk *et al.* 2018). Beyond CRISPR-Cas, the adaptive immune system in prokaryotes, the discovery of transposons and other innate immune systems has also contributed to the development of molecular biology tools (Durrant *et al.* 2024; Gao *et al.* 2020). The recently discovered and characterized bridge RNA-guided transposon system, IS110, can directly mediate sequence-specific rearrangements, such as insertion, deletion, or inversion, between two DNA molecules, providing broader possibilities for future genome editing applications (Durrant *et al.* 2024). With the advancement of bioinformatics and sequencing technologies, the discovery of these new systems is accelerating, highlighting the need for a standardized characterization protocol for these systems. We hope that this protocol can assist in the preliminary study of the emerging genome-editing tools and contribute to the research about their natural functions.
